# Transcriptome Profiling Identifies Key Regulators of Tuber Skin Color in Potato

**DOI:** 10.3390/plants14101544

**Published:** 2025-05-20

**Authors:** Boshu Li, Shuo Wang, Jun Hu, Liping Jin, Jianfei Xu

**Affiliations:** 1 College of Horticulture, Shanxi Agricultural University, Jinzhong 030801, China; lbz956889@163.com; 2 State Key Laboratory of Vegetable Biobreeding, Key Laboratory of Biology and Genetic Improvement of Tuber and Root Crop of Ministry of Agriculture and Rural Affairs, Institute of Vegetables and Flowers, Chinese Academy of Agricultural Sciences, Beijing 100081, China; wangshuo202099@163.com (S.W.); hujun@caas.cn (J.H.); jinliping@caas.cn (L.J.)

**Keywords:** potato, tuber skin color, transcriptome analysis, anthocyanin, *StMYB3*

## Abstract

The color of tuber skin exhibits remarkable diversity in potato (*Solanum tuberosum* L.) and is intricately associated with variance in anthocyanin accumulation across different varieties. The regulatory mechanisms governing this phenomenon are poorly understood. In this study, we identified a natural, yellow-skinned variant (Z28M) from the red-skinned tetraploid variety, Zhongshu 28 (Z28W), using simple sequence repeat (SSR) molecular marker amplification and trait observation. The transcriptional regulatory mechanisms underlying tuber skin color variation were investigated by analyzing anthocyanin profiles and transcriptomic data at the developmental and maturation stages. Ultra-performance liquid chromatography (UPLC-QTOF-MS) analysis indicated markedly reduced levels of pelargonidin and peonidin in Z28M compared with those in Z28W. Transcriptome profiling identified 1858 differentially expressed genes between Z28W and Z28M, with significant enrichment in the flavonoid and phenylpropanoid biosynthetic pathways. Weighted gene co-expression network analysis indicated a red-skinned associated module, MEred, encompassing key anthocyanin biosynthetic genes co-expressed with the transcription factor, *StMYB3*, which exhibited substantially higher expression in Z28W than in Z28M. K-means clustering indicated coordinated expression patterns among *StCHS*, *StDFR*, and *StMYB3*, suggesting transcriptional co-regulation. Collectively, these results highlight *StMYB3* as a pivotal regulator of anthocyanin biosynthesis and a contributor to the tuber skin color divergence observed between Z28W and Z28M.

## 1. Introduction

Potato (*Solanum tuberosum* L.), an annual herbaceous species of the Solanaceae family, is one of the world’s most important food crops. It is valued not only for its yield and adaptability, but also for its rich nutritional profile, including high levels of starch, dietary fiber, vitamins, and essential trace elements. Pigmented cultivars accumulate substantial amounts of anthocyanins, which are natural, nontoxic flavonoid pigments with antioxidant, anticancer, and anti-atherosclerotic activities [1].

Anthocyanins are water-soluble pigments classified as secondary metabolites of the flavonoid family and are responsible for the blue, purple, and red coloration of various fruits, vegetables, and flowers. Due to their physicochemical instability, anthocyanins typically exist as stable glycosides [2]. Their content is positively correlated with pericarp color, with low levels producing yellow or yellow-green hues, and high levels resulting in red or purple pigmentation. Over 20 types of anthocyanins have been identified in plants, of which pelargonidin, cyanidin, delphinidin, peonidin, petunidin, and malvidin are predominant in plants [3,4,5]. Anthocyanin biosynthesis is a conserved process that occurs via the phenylpropanoid and flavonoid biosynthetic pathways [6] and involves three main stages: skeleton formation, precursor synthesis, and glycoside modification [7]. The process is catalyzed by a series of enzymes, including phenylalanine ammonia-lyase (PAL) [8], cinnamate 4-hydroxylase (C4H) [9], 4-coumarate-CoA ligase (4CL) [10], chalcone synthase (CHS) [11], chalcone isomerase (CHI) [12], flavanone 3-hydroxylase (F3H) [13], flavonoid 3′-hydroxylase (F3′H), flavonoid 3′,5′-hydroxylase (F3′5′H) [14], dihydroflavonol 4-reductase (DFR) [15], and anthocyanidin synthase (ANS) [16], followed by glycosylation and methylation to generate stable derivatives such as peonidin glucoside.

Anthocyanin biosynthesis is tightly controlled at the transcriptional level, primarily by the MYB, bHLH, and WD40 transcription factors, which form MBW complexes to activate or repress target genes [17]. In *Arabidopsis*, R2R3-MYB proteins regulate key structural genes such as *DFR*, *ANS*, and UDP-glucose: flavonoid 3-O-glucosyltransferase (*UFGT*) [18], whereas early biosynthetic genes are modulated by *MYB11*, *MYB12*, *MYB111*, and *MYB75* [19]. Similarly, in potato, *StMYB200* and *StMYB210* cooperatively regulate anthocyanin biosynthesis by activating structural genes and interacting with *StbHLH1*, thereby promoting pigment accumulation in tuber flesh [20]. Additional regulators, including COP1, JAZ, NAC, SPL, and WRKY, participate in the fine-tuning of this pathway [18]. Tuber skin coloration in potatoes is primarily determined by the accumulation of anthocyanins, particularly pelargonidin and petunidin, in red-skinned cultivars [21]. Tissue-specific pigment accumulation is governed by the R, P, and D loci. Specifically, the R locus regulates the expression of *DFR*, promoting the biosynthesis of pelargonidin-based anthocyanins responsible for red pigmentation. The P locus encodes *F3′5′H*, which facilitates the synthesis of peonidin- and petunidin-based anthocyanins contributing to violet pigmentation [22]. The D locus is involved in regulating the expression of anthocyanin biosynthetic genes specifically in the tuber skin, thereby controlling pigment accumulation in the epidermal tissues [23]. The I locus, identified in diploid potatoes, encodes an R2R3-MYB transcription factor that modulates epidermal-specific expression of anthocyanin biosynthetic genes [24] and is considered functionally equivalent to the D locus in tetraploid cultivars. Although pigment formation in tuber skin and flesh is generally controlled by conserved pathways, tissue-specific transcription factors play a critical role in establishing distinct pigmentation patterns [25].

Plants in natural environments are susceptible to mutagenesis as a result of a variety of factors. From a biological perspective, these mutations may be induced by viruses and bacteria [26]. Environmental stimuli, such as temperature fluctuations, light exposure, and soil conditions, have also been shown to contribute to genetic variation [27]. In addition, chemical and physical mutagens, such as antibiotics, alkylating agents, and ionizing radiation, are widely recognized for their ability to induce heritable changes and are frequently used by breeders to develop new varieties [28]. Extensive mutagenesis studies have been conducted in model and crop species such as *Arabidopsis thaliana*, maize, and rice. In most cases, mutation identification relies on genome sequencing, quantitative trait loci (QTL) mapping, association analysis, and transcriptome analysis [26]. However, research on natural potato mutants remains limited, as most mutants are generated using targeted mutagenesis techniques [29]. For instance, a red-fleshed potato mutant was obtained through the in vitro regeneration of wild-type purple potatoes during tuber color variation studies [30].

In the present study, the red-skinned cultivar, Z28W, and its yellow-skinned mutant, Z28M, were used to investigate the transcriptional regulatory mechanisms governing tuber skin color formation. Anthocyanin content was quantified at the developmental and maturation stages, accompanied by transcriptome sequencing. A series of integrated analyses, including phenotypic characterization, ultra-performance liquid chromatography–mass spectrometry (UPLC-QTOF-MS)-based anthocyanin profiling, simple sequence repeat (SSR) fingerprinting, transcriptomic analysis, weighted gene co-expression network analysis (WGCNA), K-means clustering, promoter cis-element prediction, and quantitative reverse transcription polymerase chain reaction (RT-qPCR) validation, were performed to determine the regulatory roles of transcription factors involved in anthocyanin biosynthesis. These findings offer new insights into the molecular basis of potato skin pigmentation and provide a theoretical framework for the genetic improvement of pigmented cultivars with enhanced nutritional and commercial value.

## 2. Results

### 2.1. Phenotypic and Genotypic Characterization

**Figure 1:**
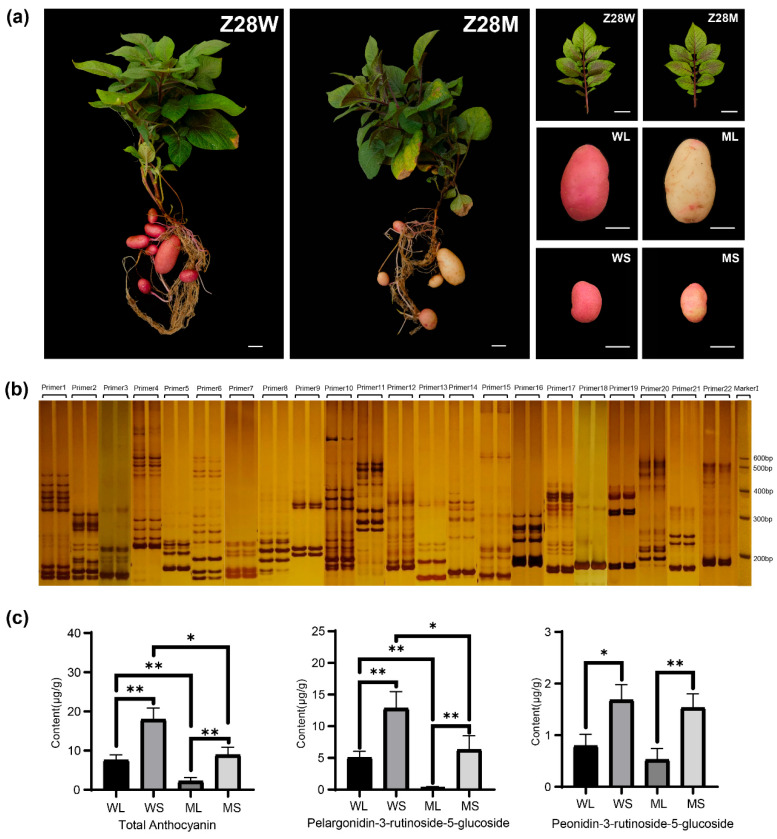
Phenotypic characteristics, SSR fingerprinting, and anthocyanin profiles of Z28W and Z28M potato lines. (**a**) Phenotypes of Z28W and Z28M (scale bar = 2 cm). Whole plants, leaves, mature tubers (WL: wild type at maturity; ML: mutant at maturity), and developing tubers (WS: wild type at developing stage; MS: mutant at developing stage). (**b**) Fingerprinting SSR markers. For each primer pair, the left lane corresponds to Z28W and the right lane to Z28M. “Marker” indicates the DNA size standard. (**c**) Differences in anthocyanin fractions in potato skin between the wild type and mutant. * Indicates significant differences; * *p* < 0.05, ** *p* < 0.01.

Phenotypic comparison between the wild-type, Z28W, and mutant, Z28M, indicated no discernible differences in aboveground traits, including leaf color, stem pigmentation, plant height, stem thickness, and branching patterns (Figure 1a). However, clear differences were observed in tuber skin coloration. Mature Z28W tubers (WL; Figure 1a) and developing tubers (WS) displayed uniform red skin that was readily visible to the naked eye. In contrast, the mature tubers of Z28M (ML) were predominantly yellow, with a faint reddish tint near the bud eye, whereas the developing tubers (MS) exhibited irregular yellow and light red patches.

To assess the potential genomic divergence, SSR-based fingerprinting was performed using markers distributed across all 12 potato chromosomes. The resulting banding patterns were nearly identical for Z28W and Z28M, indicating no detectable genomic variation at the SSR level (Figure 1b).

These findings suggest that the observed differences in skin color are more likely attributable to changes in the regulatory pathways governing anthocyanin biosynthesis or accumulation. To test this hypothesis, anthocyanin content and composition were quantified across the four tuber skin groups. Five anthocyanins were identified (Table S1), of which pelargonidin and peonidin were predominant, with pelargonidin most abundant. At both developmental stages, Z28M exhibited substantially reduced anthocyanin levels compared with Z28W (Figure 1c), indicating compromised anthocyanin biosynthesis in the mutant.

### 2.2. Transcriptome Profiling and Functional Enrichment Analysis of Differentially Expressed Genes (DEGs)

**Figure 2:**
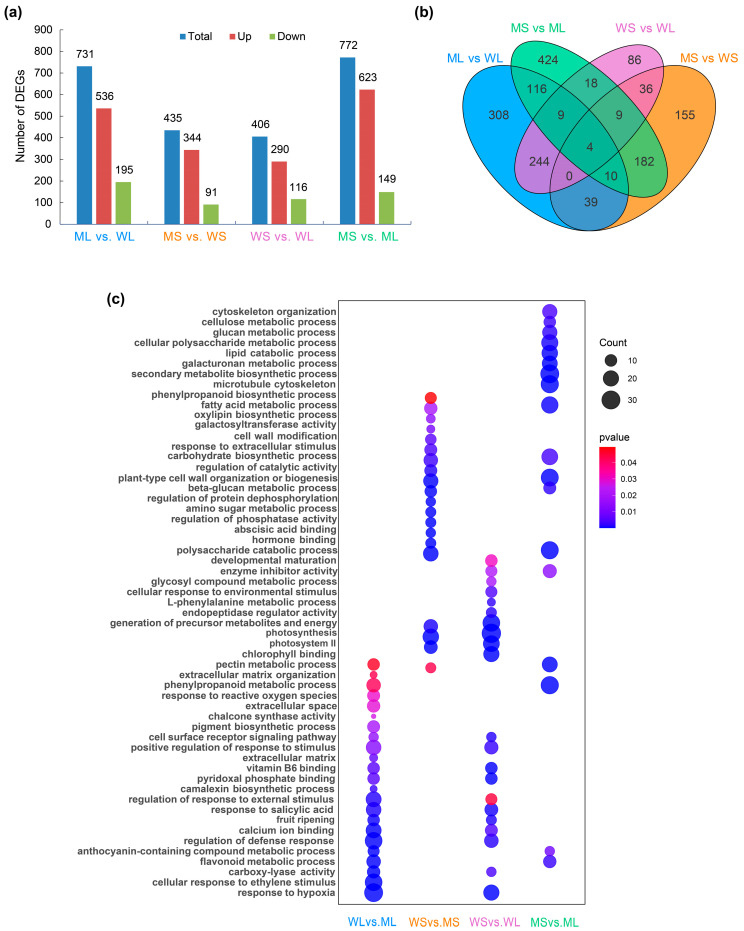
Differentially expressed gene (DEG) analysis among different tuber samples. (**a**) The number of DEGs identified in four pairwise comparisons: WL vs. ML, WS vs. MS, WS vs. WL, and MS vs. ML; blue bars represent total DEGs, red represents upregulated genes, and green represents downregulated genes. (**b**) A Venn diagram showing the overlap and specificity of DEGs among the four comparisons. (**c**) Gene Ontology (GO) enrichment analysis of DEGs in each comparison. The size of each dot indicates the number of genes enriched in the corresponding GO term, and the color represents the adjusted *p*-value.

To elucidate the molecular basis of tuber skin color variation, transcriptome sequencing was conducted on four groups of potato skin samples: WL, WS, ML, and MS. A total of 1858 unique DEGs were identified across all pairwise comparisons. The largest number of DEGs, comprising 623 upregulated and 149 downregulated genes, was detected in the MS vs. ML comparison. The ML vs. WL comparison yielded 731 DEGs, including 536 upregulated and 195 downregulated genes. In the MS vs. WS group, 435 DEGs were identified, of which 344 were upregulated and 91 were downregulated (Figure 2a).

Venn diagram analysis was performed for four pairwise comparisons. Subsequent analyses focused on WL vs. ML, WS vs. MS, and MS vs. ML, which involved the mutant line and showed clear differences in tuber skin color. The WS vs. WL comparison was included for completeness but not emphasized, as no significant color change was observed during wild-type development. Partial intersections were observed (Figure 2b), indicating shared regulatory components, including genes potentially involved in anthocyanin biosynthesis.

GO enrichment analysis indicated distinct functional signatures across the comparisons (Figure 2c). In the ML vs. WL group, DEGs were significantly enriched in the flavonoid biosynthetic (GO:0009813) and phenylpropanoid metabolic processes (GO:0009698), both of which are associated with secondary metabolism and the production of flavonoid-derived compounds. In the MS vs. WS group, the enriched terms included photosynthetic membrane (GO:0034357) and external encapsulating structure organization (GO:0045226), reflecting developmental differences in photosynthetic activity and cell wall dynamics. The MS vs. ML group exhibited the most diverse enrichment patterns, with DEGs involved in secondary metabolite biosynthesis (GO:0044550), phenylpropanoid catabolism (GO:0046271), flavonoid glucuronidation (GO:0052696), and anthocyanin-containing compound biosynthesis (GO:0009718). In contrast, the DEGs in the WS vs. WL comparison were primarily enriched for fruit maturation (GO:0010154) and photosystem II assembly (GO:0010207), indicating regulatory shifts during early tuber development.

### 2.3. Identification of Tuber Skin Color-Associated Modules via Weighted Gene Co-Expression Network Analysis (WGCNA)

**Figure 3:**
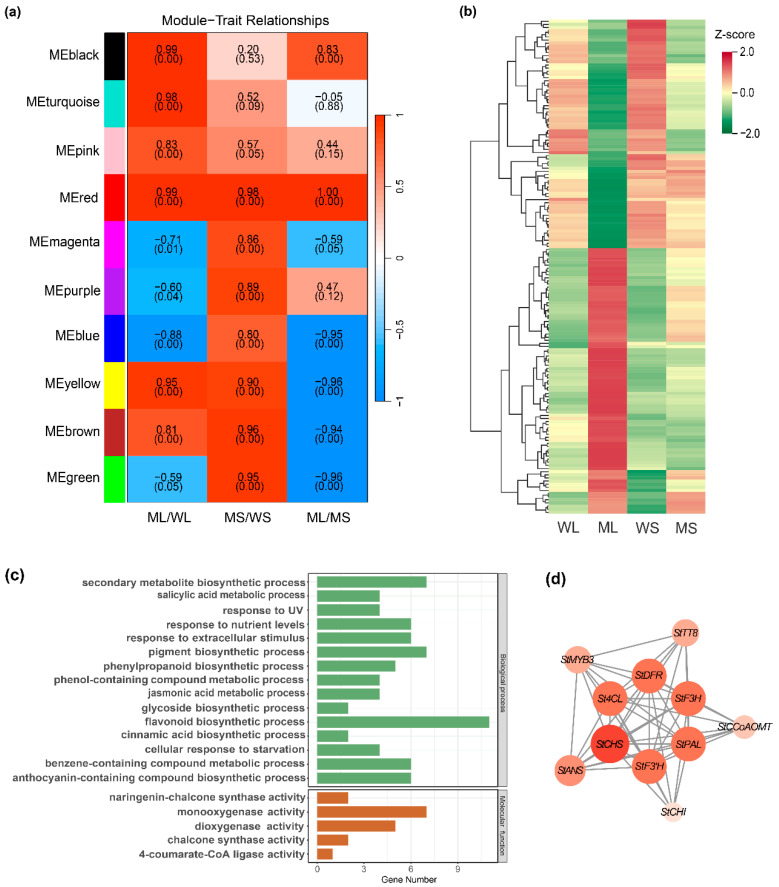
Weighted gene co-expression network analysis-based identification and characterization of an anthocyanin-related co-expression module. (**a**) A correlation heatmap between gene co-expression modules and sample traits. Colors represent the strength and direction of correlation, with *p*-values shown below each coefficient. (**b**) An expression heatmap of differentially expressed genes within the MEred module across WL, ML, WS, and MS samples. (**c**) Gene Ontology enrichment analysis of genes in the MEred module. (**d**) A co-expression network of anthocyanin-related genes within the MEred module.

WGCNA was conducted using the full set of 1858 DEGs, resulting in ten distinct co-expression modules (Figure 3a). Of these, the MEred module exhibited the strongest correlation with the three pairwise comparisons that showed the most pronounced color differences: MS vs. ML, ML vs. WL, and MS vs. WS. This module comprised 158 genes. Gene expression values were normalized using Z-score transformation for downstream visualization (Figure 3b; Table S4).

GO enrichment analysis was performed on member genes to investigate the functional relevance of the MEred module. The significantly enriched terms were visualized as bar plots (Figure 3c), highlighting the biological processes and molecular functions associated with pigment metabolism. The enriched biological processes included anthocyanin-containing compound biosynthesis (GO:0009718), flavonoid biosynthesis (GO:0009813), benzene-containing compound metabolism (GO:0042537), pigment biosynthesis (GO:0046148), chalcone synthase activity (GO:0102128), phenylpropanoid biosynthesis (GO:0009699), and secondary metabolite biosynthesis (GO:0044550). In terms of molecular functions, significant enrichment was observed for dioxygenase (GO: 0051213) and 4-coumarate-CoA ligase (GO: 0016207) activities, both of which are integral to flavonoid and anthocyanin production, respectively. These results suggest that the MEred module plays a regulatory role in tuber skin pigmentation.

A co-expression network of the MEred module was constructed and visualized using Cytoscape_v3.10.3, with key anthocyanin biosynthetic genes highlighted based on their pathway relevance (Figure 3d). The resulting network consisted of 11 gene nodes, including 2 transcription factors, *StMYB3* and *StTT8*. StCHS, a pivotal enzyme initiating the flavonoid biosynthetic pathway, exhibited the highest connectivity and served as a central hub. Other key structural genes included *StANS*, which catalyzes the final step in anthocyanin formation; *StF3′H*, which modulates anthocyanin type and coloration; and StPAL, a gateway enzyme in the phenylpropanoid pathway. Collectively, these genes constitute a coordinated regulatory network that likely contributes to anthocyanin accumulation in potato tuber skin.

### 2.4. Expression Clustering Reveals an Anthocyanin-Associated Gene Module

**Figure 4:**
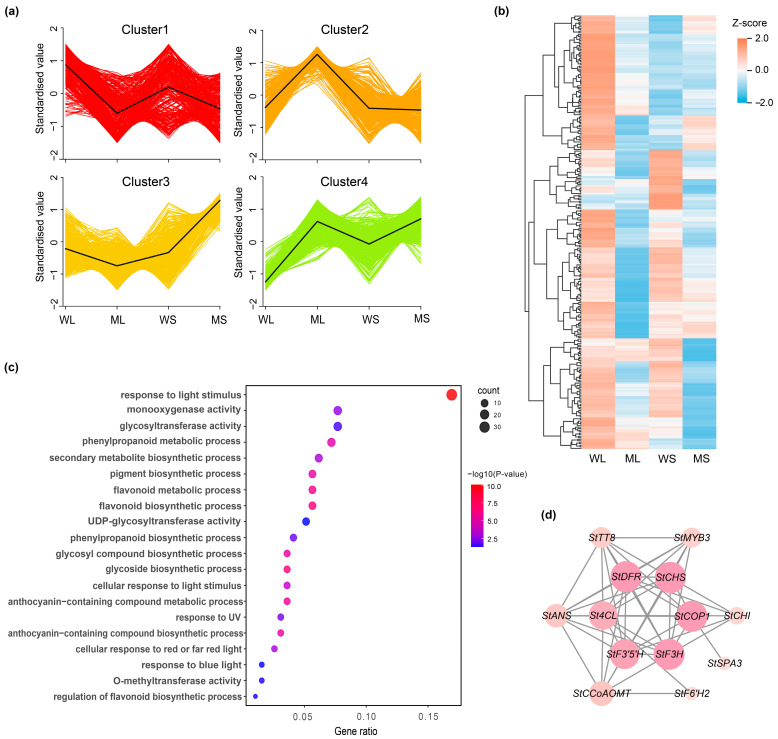
K-means clustering analysis of differentially expressed genes (DEGs). (**a**) K-means clustering of gene expression profiles across WL, ML, WS, and MS samples, resulting in four distinct clusters with characteristic patterns. (**b**) Heatmap showing standardized expression of genes in Cluster 1. (**c**) Gene Ontology enrichment analysis of Cluster 1 genes. Dot size represents gene count, and color indicates statistical significance. (**d**) Co-expression network of anthocyanin-related genes identified in Cluster 1.

K-means clustering analysis was used to group the DEGs based on their shared expression patterns. The resulting clusters exhibited distinct transcriptional profiles (Figure 4a; Table S5). Cluster 1 contained 287 genes with high expression in the Z28W samples, Cluster 2 included 295 genes predominantly expressed in ML, Cluster 3 consisted of 768 genes with elevated expression in MS, and Cluster 4 comprised 512 genes that were also upregulated in Z28W. Cluster 1 was enriched with genes associated with anthocyanin biosynthesis. Heatmap visualization indicated that most genes in this cluster were strongly expressed in WL and WS but showed substantially reduced expression in ML and MS (Figure 4b), consistent with the observed decrease in anthocyanin content in the mutant.

GO enrichment analysis of Cluster 1 highlighted multiple biological processes related to pigment metabolism (Figure 4c), including flavonoid biosynthesis and phenylpropanoid metabolism. In addition, genes involved in light signaling, such as responses to blue, red, and ultraviolet light, were significantly enriched, along with those related to enzymatic activity, particularly glycosyltransferases and methyltransferases. These results suggest that Cluster 1 genes participate in a coordinated regulatory network involved in pigment biosynthesis, which is potentially modulated by both intrinsic developmental cues and environmental light signals.

To explore potential functional relationships, genes within Cluster 1 related to anthocyanin biosynthesis were submitted to the STRING database for protein–protein interaction (PPI) network analysis (Figure 4d). The resulting network featured several key structural genes, including *StCHS*, *StDFR*, *StF3′H*, *StF3H*, and *StANS*, alongside two transcription factors, *StMYB3* and *StTT8*. Among these, *StCHS* and *StDFR* exhibited the highest connectivity, emphasizing their central roles in the anthocyanin biosynthetic pathway. *StMYB3* displays multiple predicted interactions with structural genes, reinforcing its role as an upstream regulator. Additionally, light-responsive genes, such as *StCOP1* and *StSPA3,* were also present in the network, indicating a potential regulatory link between anthocyanin accumulation and light signaling pathways.

### 2.5. Gene Expression Changes in the Anthocyanin Biosynthetic Pathway

**Figure 5:**
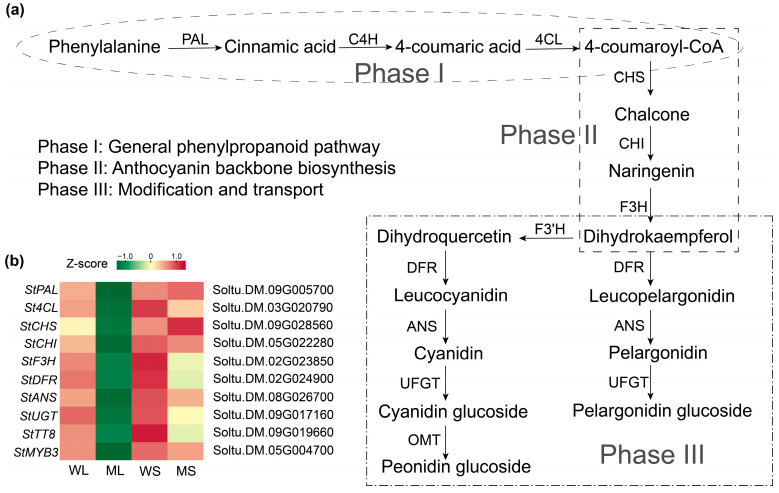
Anthocyanin biosynthetic pathway and gene expression profiles in wild-type and mutant potato tuber skins. (**a**) A schematic representation of the anthocyanin biosynthesis pathway in potato. The upstream and intermediate steps follow the conserved phenylpropanoid and flavonoid biosynthetic routes, whereas the final anthocyanin products were drawn based on compounds identified in this study. (**b**) A heatmap showing the transcript abundance of key structural and regulatory genes in WL, ML, WS, and MS samples. Expression levels are shown as Z-scores.

Anthocyanin biosynthesis is primarily regulated by structural genes controlled by the MBW transcriptional complex. Based on the types of anthocyanins identified in the potato skin, a biosynthetic pathway was constructed to illustrate the underlying regulatory framework (Figure 5a). Transcriptome analysis indicated distinct expression patterns of both structural genes and transcriptional regulators in the four sample groups. Core structural genes, including homologs of *StPAL*, *StCHS*, *StCHI*, *StF3H*, *StDFR*, and *StANS*, along with specific MYB and bHLH family transcription factors, were differentially expressed between Z28W and Z28M (Figure 5b). These results suggest that transcriptional alterations in the mutant tuber epidermis affect multiple steps in anthocyanin biosynthesis, from precursor generation and scaffold formation to final pigment modification.

### 2.6. Quantitative Real-Time PCR Validation of Gene Expression

**Figure 6:**
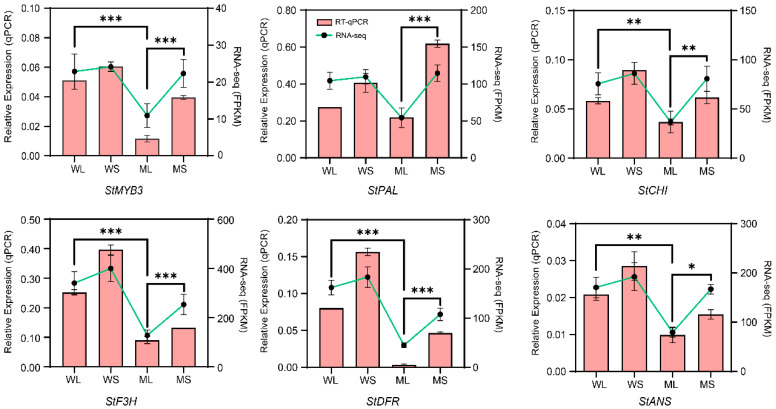
Quantitative reverse transcription polymerase chain reaction (RT-qPCR) validation of anthocyanin biosynthesis-related gene expression. Relative expression levels of *StMYB3*, *StPAL*, *StCHS*, *StF3H*, *StDFR*, and *StANS* in WL, WS, ML, and MS samples. Pink bars represent RT-qPCR results and green lines show RNA-seq expression (FPKM). Error bars indicate standard deviations from three biological replicates (*n* = 3). Statistical significance was calculated using Student’s *t*-test. Significant differences in RT-qPCR expression between WL and ML and WS and MS are indicated by * *p* < 0.05, ** *p* < 0.01, and *** *p* < 0.001.

To confirm the expression patterns of candidate genes involved in anthocyanin biosynthesis, several structural genes and the transcription factor *StMYB3*, a key regulator identified in the co-expression network, were selected for RT-qPCR validation. The cDNA synthesized from the WL, WS, ML, and MS samples was used as the amplification template, and gene expression levels were compared with the corresponding transcriptome data (Figure 6). The results showed that *StMYB3* and the structural genes related to anthocyanin biosynthesis, including *StPAL*, *StCHI*, *StF3H*, *StDFR*, and *StANS*, exhibited significant differences in expression among the samples. *StMYB3* showed the highest expression level in WS and the lowest expression level in MS. Most structural genes displayed higher expression in the wild type, particularly during the developing stage, while their expression levels were generally lower in the mutant samples. The expression patterns determined by RT-qPCR were consistent with the transcriptome sequencing results, further confirming these expression patterns.

### 2.7. Prediction of StMYB3 Binding Sites in Anthocyanin Biosynthetic Gene Promoters

**Figure 7:**
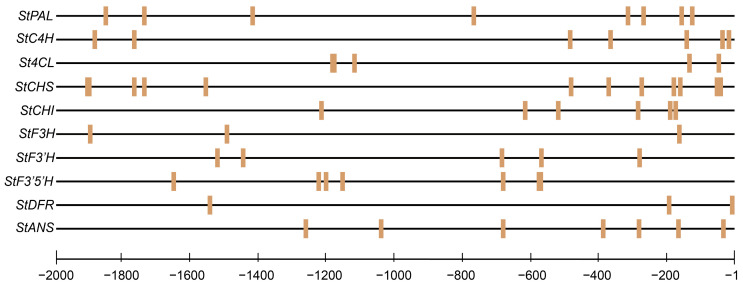
Predicted *StMYB3* binding sites in the promoter regions of key anthocyanin biosynthetic genes based on JASPAR analysis.

Binding site prediction was conducted using the JASPAR database, specifically based on the *StMYB3* recognition motif (MA1038), to identify potential target sites within the 2000 bp upstream promoter regions of anthocyanin biosynthetic genes. The analysis revealed that several structural genes—including *StPAL*, *StCHS*, *StCHI*, *StF3′H*, *StF3′5′H*, *StDFR*, and *StANS*—contained high-confidence MYB-binding motifs. These elements were unevenly distributed along the promoter regions, with certain genes exhibiting multiple binding sites. Several motifs were located in the regions less than 500 bp from the transcription start site, suggesting a potentially stronger regulatory effect of *StMYB3* on transcriptional initiation (Figure 7).

## 3. Discussion

The mutant tuber epidermis exhibited lower anthocyanin content than the wild-type material at the same developmental stage, as reflected in both phenotypic traits and anthocyanin quantification results. This suggests that anthocyanin biosynthesis is hindered in this mutant. The mutant, Z28M, was derived from field-grown Z28W plants, and SSR fingerprinting showed no detectable genomic variation. Although this finding suggests potential epigenetic or small-scale genetic changes, further genome sequencing is required to identify the exact mutation. This study investigated the differential mechanisms of anthocyanin biosynthesis in red- and yellow-skinned potato cultivars through a comprehensive analysis that included phenotypic characterization, transcriptomic profiling, and anthocyanin quantification. Transcriptomic analysis identified key DEGs in the anthocyanin biosynthesis pathway, indicating significantly altered expression profiles in the yellow-skinned mutants. The reduced anthocyanin content in the mutants was associated with the downregulation of critical biosynthetic genes, including *StCHS*, *StDFR*, and *StANS*, compared to wild-type potatoes. GO enrichment analysis further highlighted a decline in anthocyanin-related biological processes in the yellow-skinned variants. The co-expression network indicated that the structural genes involved in anthocyanin biosynthesis were regulated by the MYB and bHLH transcription factors, suggesting that the disrupted regulation of these factors may contribute to the observed discoloration.

Analysis of *StMYB3* (Soltu.DM.05G004700), referred to as *MYB3* based on homologous gene comparison, indicated a frequent presence in the co-expression network. The expression pattern was positively correlated with the anthocyanin content. Previous studies have shown that *StMYB3* positively regulates anthocyanin synthesis, consistent with our experimental findings [31]. The expression of *StMYB3* was further validated by RT-qPCR, and the results are consistent with the transcriptome data (Figure 6). These results suggest that reduced expression of *StMYB3* may play a key regulatory role in potato peel discoloration in mutants. Regulatory interactions govern flavonoid synthesis and anthocyanin accumulation [32]. Similar regulatory mechanisms have been reported in other species, including tomatoes [33] and strawberries [34], where MYB factors directly control anthocyanin biosynthesis genes.

Bioinformatic analysis identified several *StMYB3* binding sites in the promoter regions of the *StDFR* and *StANS* genes, suggesting that *StMYB3* has the potential to regulate the expression levels of synthetic genes, which can be further elucidated using dual-luciferase reporter, yeast one-hybrid, and chromatin immunoprecipitation assays. These findings not only shed light on the pigmentation mechanism in tuber skin but also identify candidate regulators for potential molecular breeding of anthocyanin-enriched potato cultivars.

This study demonstrated a direct link between reduced anthocyanin accumulation in mutant potato skin and the downregulation of structural genes involved in the biosynthetic pathway. Of these regulatory factors, *StMYB3* likely plays a pivotal role in the modulation of gene expression. Future studies should focus on the functional validation of *StMYB3*, such as through overexpression or CRISPR-Cas9-mediated knockout in potatoes, to confirm its regulatory function in anthocyanin biosynthesis. By integrating phenotypic, transcriptomic, and promoter analyses, this study advances our understanding of the molecular mechanisms underlying epidermal pigmentation in tubers. These findings provide valuable genetic insights into potato skin color regulation and offer potential strategies for enhancing anthocyanin content, with implications for improving both the nutritional quality and commercial appeal of pigmented potato cultivars.

## 4. Materials and Methods

### 4.1. Plant Materials and Growth Conditions

The potato cultivar, ‘Zhongshu 28’, was provided by the Potato Research Laboratory at the Institute of Vegetables and Flowers of the Chinese Academy of Agricultural Sciences. The wild-type line, Z28W, had red skin and white flesh, whereas the mutant line, Z28M, derived from field-grown plants, had yellow skin and white flesh. The mutant was propagated using shoot tip detoxification and clonal multiplication [35]. Seedlings of both genotypes, grown for 4 w, were transplanted into plastic pots measuring 10 × 8 cm and cultivated in a greenhouse under controlled conditions at 18–22 °C, with a photoperiod of 8 h light and 16 h dark. Tubers were harvested 70 d after transplantation and divided into two developmental stages based on the transverse diameter: mature tubers measuring 6–7 cm, and developing tubers measuring 2–3 cm. Skin samples approximately 1 mm thick were collected from the middle section of each tuber. Three biological replicates were prepared for each sample group by pooling skin tissues from multiple tubers. These groups were named wild-type mature (WL), wild-type developing (WS), mutant mature (ML), and mutant developing (MS). All samples were immediately frozen in liquid nitrogen and stored at −80 °C until further analysis.

### 4.2. DNA Extraction and SSR Analysis

Fresh, young leaves weighing 2 g were collected for genomic DNA extraction using a modified cetyltrimethylammonium bromide (CTAB) method [36]. The extracted DNA was diluted to a concentration of 30 ng·mL^−1^ and stored at −20 °C until further use. Simple sequence repeat (SSR) analysis, a widely applied technique for assessing plant genetic diversity [37], was performed using primer pairs synthesized by HuaDa Biotech Company in Beijing, China. These primers spanned all 12 potato chromosomes and can identify approximately 149 potato varieties based on their genomic profiles [38]. Polymerase chain reaction (PCR) amplification was carried out in a 10 µL reaction system consisting of 1.0 µL DNA template, 0.5 µL each of forward and reverse primers at 10 µM, 5.0 µL of Vazyme 2× 3G Taq Master Mix for polyacrylamide gel electrophoresis (PAGE), and 3.0 µL of double-distilled water.

The touchdown PCR program included initial denaturation at 95 °C for 5 min, followed by 35 cycles of 30 s at 95 °C, 30 s of annealing starting at 56 °C, and 30 s of extension at 72 °C, with a final extension at 72 °C for 7 min. Amplified SSR products were analyzed using PAGE to verify sample identity and rule out species contamination. The SSR primers used in this study are listed in Supplementary Table S2.

### 4.3. Determination of Anthocyanin Type and Content

The anthocyanin content in potato skin samples was determined strictly following the method described by Wang et al. (2020) [39], with no modifications. Each group contained three biological replicates, with each replicate consisting of 0.2 g of freeze-ground potato skin powder. The fresh samples were ground in liquid nitrogen to prevent pigment degradation. The powdered tissue was extracted with 4 mL methanol containing 10% formic acid (volume ratio 9:1) in amber glass tubes. An internal standard (15 µg) of peonidin-3-glucoside chloride was added to each tube to ensure accurate quantification. Samples were incubated at 4 °C for 12 h under constant agitation. Following extraction, the mixtures were centrifuged at 3900 rpm for 20 min and the resulting supernatants were transferred to fresh tubes. The supernatants were evaporated under nitrogen and the dried residues were dissolved in 1 mL of methanol/formic acid (9:1). The solutions were filtered through a 0.22 µm nylon membrane prior to analysis. Anthocyanins were quantified using ultra-performance liquid chromatography coupled with quadrupole time-of-flight mass spectrometry (UPLC-QTOF-MS). Light absorbance was monitored at 535 nm, and the total anthocyanin content was calculated based on the peak area of the internal standard. The analysis was performed on a Waters ACQUITY UPLC I-Class system equipped with a Xevo G2-XS QTOF mass spectrometer and a PDA Lambda detector. Chromatographic separation was achieved using a C18 column (2.1 mm × 100 mm, particle size 1.7 μm), with a mobile phase composed of acetonitrile and water containing 5% formic acid.

### 4.4. Differentially Expressed Gene (DEG) Expression and Gene Ontology (GO) Enrichment Analyses

The cDNA libraries were constructed and sequenced on an Illumina platform by Metware Biotechnology Co., Ltd. (Wuhan, China). Differential gene expression analysis was performed using DESeq2, with *p*-values adjusted for multiple testing using the Benjamini–Hochberg method to control the false discovery rate (FDR). Genes with an absolute |log₂Fold Change| ≥ 1 and FDR < 0.05 were defined as differentially expressed. GO enrichment analysis was performed using the ClusterProfiler package (version 4.6.0) [40]. GO terms with *p* < 0.05 were considered significantly enriched.

### 4.5. Gene Co-Expression Analysis and Network Visualization

DEGs from all samples were subjected to weighted gene co-expression network analysis using the WGCNA package in R [41]. The analysis was performed with the following parameters: fragments per kilobase of transcript per million mapped reads (FPKM) coefficient of variation ≥ 0.5, soft-thresholding power set to 11, and a minimum module size of 50. The resulting gene modules were used to construct co-expression networks, which were visualized using Cytoscape (version 3.10.3) [42].

### 4.6. K-Means Clustering and Correlation Network Analysis of DEGs

DEGs were clustered based on their FPKM values using the Mfuzz package in R [43]. To ensure data reliability, the average FPKM values of three biological replicates were used for each gene. Cluster 1 was selected for the subsequent correlation network analysis. DEG filtering was performed using the following criteria: FPKM > 1 and expression fold change > 2.0 or <0.5. Genes with *p*-values below 0.05 were mapped to their corresponding protein names and submitted to the STRING database (https://string-db.org/) for protein–protein interaction (PPI) analysis. The resulting interaction networks were visualized using Cytoscape (version 3.10.3) [42].

### 4.7. Quantitative Real-Time PCR (RT-qPCR) Analysis

Total RNA was extracted from powdered tuber skin samples using the RNAprep Pure Plant Kit (Tiangen, Beijing, China) according to the manufacturer’s instructions. First-strand cDNA was synthesized using the HiScript Reverse Transcriptase (Vazyme, Nanjing, China). Six DEGs were selected for RT-qPCR validation, using *EF1α* [44] as the internal reference. Each assay was conducted using three biological replicates and three technical replicates per sample to ensure reliability. Gene nomenclature was based on *Arabidopsis thaliana* orthologs, as annotated in TAIR (https://www.arabidopsis.org/). RT-qPCR was performed using a two-step protocol on a LightCycler 480 II system (Roche, Switzerland). Relative expression levels were calculated using the 2^−ΔΔCT^ method [45]. The primer sequences are listed in Supplementary Table S6, and the corresponding gene identifiers are provided in Supplementary Table S5.

### 4.8. Promoter Sequence Extraction and Cis-Element Prediction

To further investigate the potential target genes of *StMYB3*, ten structural genes involved in anthocyanin biosynthesis were selected based on differential expression and co-expression network analyses. These genes included *StPAL*, *StC4H*, *St4CL*, *StCHS*, *StCHI*, *StF3H*, *StF3′H*, *StF3′5′H*, *StDFR*, and *StANS*. The promoter sequences, defined as the 2000 bp upstream regions from the start codon of each gene, were retrieved from the Spud DB database. Putative *StMYB3* binding sites were predicted using the JASPAR database. Only high-confidence motifs with prediction scores ≥ 80 were retained for downstream analysis. The tools and resources used in the analysis are listed in Supplementary Table S7.

## 5. Conclusions

In this study, we investigated the regulatory mechanisms underlying tuber skin color variation in potatoes using Z28W (wild type with red skin) and Z28M (mutant with yellow skin). Integrated analyses, including phenotyping, anthocyanin profiling, transcriptome sequencing, WGCNA, and K-means clustering, indicated that anthocyanin content and key biosynthetic genes were significantly downregulated in Z28M during tuber development. In total, 1858 DEGs were identified, and the MEred module and Cluster1 showed strong correlations with skin color differences. The transcription factor, *StMYB3*, was co-expressed with multiple structural genes involved in anthocyanin biosynthesis and exhibited consistent expression patterns across analytical approaches. Sequence and phylogenetic analyses confirmed that *StMYB3* encoded a nuclear-localized R2R3-MYB protein closely related to known anthocyanin regulators in *Arabidopsis*. These results suggest that *StMYB3* plays a key role in the regulation of anthocyanin biosynthesis and contributes to tuber skin pigmentation in potatoes. Future functional studies using in vivo assays or clustered regularly interspaced short palindromic repeats-based gene editing are required to verify the regulatory role of *StMYB3* in anthocyanin biosynthesis.

## Data Availability

The data are contained within the article and Supplementary Materials.

## Supplementary Materials

The following supporting information can be downloaded at https://www.mdpi.com/article/10.3390/plants14101544/s1: Table S1: Anthocyanin types and their contents; Table S2: Primer sequences used for PCR amplification; Table S3: Table summary of mapping results (mapping to genome); Table S4: Genes enriched by the MEred module, related to Figure 3; Table S5: The clustered group of the differently expressed genes, related to Figure 4; Table S6: A list of the quantitative real-time PCR primers used in this study; Table S7: A description of the application of online databases and bioinformatic tools.
